# Seroprevalence of measles immunoglobulin G antibodies in Bahrain: A cross-sectional study using fever and rash surveillance data (2021-2025)

**DOI:** 10.1016/j.ijregi.2026.100871

**Published:** 2026-03-07

**Authors:** Adel Alsayyad, Ebrahim Matar, Zahra Zabar, Mona Husain, Wafa Hasan, Malak Hasan, Maram Hasan, Janan Radhi, Basma Al Safar, Afaf Mohamed

**Affiliations:** 1 Family and Community Medicine Department, Arabian Gulf University, Manama, Bahrain; 2 Ministry of Health, Manama, Bahrain; 3 Public Health Directorate, Ministry of Health, Manama, Bahrain; 4 Royal Medical Services, Manama, Bahrain; 5 Royal College of Surgeons in Ireland, Busaiteen, Bahrain

**Keywords:** Measles, Seroprevalence, Bahrain, Vaccination, IgG antibodies, Surveillance

## Abstract

**Objectives:**

Measles remains a public health challenge despite effective vaccines; recent declines in coverage led to resurgent outbreaks worldwide. This study aims to estimate measles immunoglobulin (Ig)G seroprevalence in Bahrain’s population and identify immunity gaps using serological data from the national fever and rash surveillance system.

**Methods:**

We analyzed fever and rash surveillance data from Bahrain (2021-2025). Suspected measles cases were tested for measles IgG antibodies. Seroprevalence was stratified by age, nationality, vaccination status, and governorate. Overall prevalence was age-adjusted, and differences in seropositivity were tested using chi-square tests.

**Results:**

Among 1280 patients, the overall seroprevalence of measles IgG was 81.6% (95% confidence interval: 79.3-83.7%). Seropositivity differed between Bahrainis and non-Bahrainis (82.6% vs 77.3%; *P* = 0.047). There was strong evidence of differences in seroprevalence by age (*P* <0.001). Seroprevalence was 10.5% among those aged less than 6 months and dropped to 4.9% among those aged 6-12 months. Adjusted for age, seroprevalence was 94.3% (95% confidence interval: 85.7-97.9%).

**Conclusion:**

Bahrain’s age-adjusted measles seroprevalence is within herd immunity thresholds. However, vulnerabilities exist among those aged less than 18 months and non-Bahrainis. Sustained high measles, mumps, and rubella vaccine coverage and enhanced surveillance are important in maintaining measles elimination status.

## Highlights

• A seroprevalence study nested in Bahrain’s fever and rash surveillance system.
• Age-adjusted measles immunoglobulin (Ig)G seroprevalence was 94.3% (95% confidence interval 85.7-97.9).
• Seroprevalence was 10.5% under 6 months and 4.9% at 6-12 months of age.
• Non-Bahraini patients had lower measles IgG seroprevalence than Bahrainis.
• Two-dose measles vaccine recipients reached 99.6% IgG seropositivity.

## Introduction

Measles, a single-stranded RNA virus belonging to the *Paramyxoviridae* family, is among the most contagious infectious diseases, with an estimated basic reproduction number (R_0_) ranging from 12 to 18 [1,2]. From the 19^th^ century until the pre-vaccine era of the 20^th^ century, measles was endemic worldwide, resulting in approximately 200 million deaths [3]. Since the introduction of an effective measles vaccine in the 1960s, the incidence of measles cases has significantly declined [4]. The measles vaccine is highly effective; a single dose offers 93% efficacy, and two doses provide 97% efficacy [5].

The proportion of a population required to be immune to prevent infectious disease epidemics is referred to as the herd immunity threshold—a parameter used to guide national vaccination program targets and disease elimination strategies [6]. This threshold is determined by the transmissibility of the infectious agent, quantified by the basic reproduction number R₀. A widely accepted formula is that herd immunity threshold equals 1 − 1/R₀, with the assumption of random vaccination patterns and homogeneous mixing of individuals within the population [6].

For measles, with its high transmissibility, the herd immunity threshold is estimated to be between 89% and 94%, depending on the setting [7]. Although population immunity represents a composite outcome of natural infection, vaccination programs, and maternal antibodies; vaccination programs serve as the mainstay for achieving and maintaining population immunity, particularly, in settings with low natural transmission rates [8].

It is estimated that between 2000 and 2023, measles vaccines prevented around 60 million deaths [9]. Despite these successes, recent progress toward measles elimination has encountered setbacks, particularly, after the COVID-19 pandemic. In 2023, the World Health Organization (WHO) reported a drop in global vaccination coverage levels from 87% to 83%, resulting in an estimated 20% increase in the number of measles cases and more than 100,000 deaths worldwide [10]. In the United States, a country that had achieved measles elimination status in 2000, a significant multistate outbreak involving more than 300 cases occurred in early 2025 [11]. In addition, in the Eastern Mediterranean region, the incidence of measles rose by approximately 68% between 2019 and 2022 [12].

The Kingdom of Bahrain is a country in the Arabian Gulf with a land area of 786.8 km², administratively divided into four governorates: Capital, Muharraq, Northern, and Southern. The population consists of approximately 1.5 million inhabitants, of which around 46% are Bahrainis [13]. In Bahrain, the measles vaccine was introduced in 1974, when the number of reported measles cases reached up to 2000 cases annually. Since 2001, vaccine coverage estimates for first and second doses has remained above 97% nationally and above 95% at the subnational level [14]. Currently, the measles vaccine is administered as two doses of the measles, mumps, and rubella vaccine at 12 and 18 months [15]. Since 2013, Bahrain had sustained measles elimination status, which has been maintained to date [16]. However, due to the high passenger traffic and the global rise in measles cases, the risk of introduction of imported measles cases is heightened.

Upon exposure to measles via natural infection or vaccination, levels of immunoglobulin (Ig)G rise and peaks within 2 weeks, which serve as markers for previous exposure and usually persist for life [17]. Therefore, IgG levels are widely used as indicators of protective immunity [18]. Serological surveys (serosurveys) assess population immunity by measuring IgG presence within defined groups, serving as tools for evaluating vaccination program effectiveness, identifying immunity gaps, and assessing the risk of an outbreak. Study populations and methodologies in serosurveys vary widely, including general population samples, specific age groups such as infants or adults, and targeted groups such as pregnant women or occupational cohorts [17]. Some studies adopt pragmatic approaches using existing epidemiologic data and collecting blood samples from patients enrolled in fever and rash surveillance systems who are suspected of acquiring measles and rubella infections [19].

Bahrain successfully achieved measles elimination through strong immunization program achieving high routine vaccine coverage and surveillance programs [14]. However, although population immunity has been assessed using indirect indicators, the serological profile of the population has not previously been evaluated. Understanding the population immunity profile is essential for identifying susceptibility gaps and high-risk groups, assessing outbreak risk, and guiding targeted vaccination strategies [16].

This study aims to estimate measles IgG seroprevalence among the Bahraini population and identify factors associated with level of seroprevalence. A nested serosurvey will be conducted using data from the fever and rash surveillance system covering the period from January 2021 to March 2025.

## Methods

### Study design and setting

This cross-sectional study was conducted in the Kingdom of Bahrain, using data from patients enrolled in the national fever and rash surveillance system between January 2021 and March 2025. In Bahrain, measles is a notifiable disease, and all suspected cases identified in any health care facility must be reported to the Public Health Directorate within 24 hours. Case-based measles surveillance was established in 1996. A suspected measles case is defined as any patient presenting with fever and a maculopapular (non-vesicular) rash or any patient in whom a clinician suspects measles infection. All reported suspected cases are investigated by staff from the Public Health Directorate, and patients meeting the case definition undergo laboratory testing [14].

### Study sample

The study included all patients reported to have fever and maculopapular rash from January 2021 to March 2025 who were tested for measles IgG antibodies. Patients were excluded if IgG testing was not performed, the collected blood volume was insufficient, or the patient was confirmed to have measles. Confirmed cases were defined by the presence of measles-specific IgM antibodies, a four-fold increase in IgG titers, viral RNA detection via reverse transcription-polymerase chain reaction, or viral isolation, as per national guidelines. For patients with more than one measles IgG result during the study period, only the most recent result was included. No sampling strategy was applied; all individuals who met the inclusion criteria were included in the analysis.

### Laboratory testing

Patients who met the suspected measles case definition were tested for serum measles-specific IgG antibodies using an enzyme-linked immunosorbent assay. The Platelia Measles IgG kit (Bio-Rad, Ref 72686) was used for testing. This assay is reported to be 96.6% sensitive and 100% specific. IgG presence was assessed by comparing the optical density (OD) of the sample with a standardized reference range. Qualitative interpretation was based on the OD index. Samples were classified based on the ratio of the sample OD to the cut-off value. In accordance with kit instructions, ratios <0.8 were defined as negative, 0.8-1.2 as equivocal, and >1.2 as positive [20]. Positive results falling within the 1.2-1.44 range were further classified as weak positives.

### Data management

Data were extracted from routinely collected surveillance records. Variables included sex, year of birth, age at the time of sample collection, nationality, residential governorate, and vaccination status. Nationality was categorized as Bahraini or non-Bahraini. Vaccination status was categorized into four groups: below the vaccination age (less than 12 months), recipients of one dose of measles-containing vaccine (MCV), recipients of two doses of MCV, and unvaccinated individuals. Patients were classified based on serological results as either IgG-positive (including weak positive results) or IgG-negative.

### Statistical analysis

Data analysis was conducted using R software (R Foundation for Statistical Computing, Vienna, Austria) [21]. Seroprevalence of measles IgG was calculated by dividing the number of IgG-positive individuals by the total number tested, with 95% confidence intervals (CIs) computed for each estimate. Seroprevalence was then stratified by age group, sex, nationality, residential governorate, and vaccination status. The chi-square test was used to assess the strength of evidence of an association between demographic variables and IgG seropositivity.

To estimate the age-adjusted seroprevalence of measles IgG antibodies, post-stratification weighting was applied to account for differences between the sample age distribution and Bahrain's population structure [22,23]. Age-specific population proportions as of the end of 2023 were obtained from the Bahrain government open data portal, whereas corresponding sample proportions were derived from public health’s surveillance data [24]. Post-stratification weights were calculated for each age group as the ratio of the population proportion to the sample proportion. The age-adjusted seroprevalence proportion and 95% CI were estimated using the “survey” package in R, with the logit method for variance estimation [22].

### Ethical approval

The study was conducted in accordance with the principles of the Declaration of Helsinki. Routine surveillance data were extracted and anonymized before analysis. Ethical approval was obtained from the Research Ethics Committee of the Ministry of Health, Bahrain (AUPH-2025-H-00081).

## Results

### Study sample

**Figure 1:**
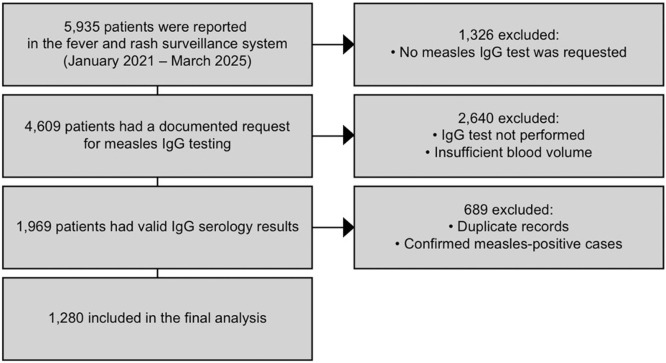
Flowchart of the inclusion process for the 1280 enrolled patients tested for measles IgG. The flowchart illustrates the inclusion and exclusion criteria applied to patients enrolled in the national fever and rash surveillance system, resulting in the final study population of 1280 patients with validated measles IgG test results. Ig, immunoglobulin.

Between January 2021 and March 2025, the Public Health Directorate received a total of 5935 notifications of patients with history of fever and rash from primary health care centers and secondary care hospitals. Among these, 4609 measles IgG tests were requested, of which 1969 results were validated (positive, weak positive, or negative for IgG). After excluding previous results for patients with multiple results, a total of 1280 distinct patients were enrolled in the study (Figure 1).

Of the 1280 patients enrolled, 724 (56.6%) were males and 1020 (79.7%) were Bahrainis. Most of the patients were residents of the Northern governorate (n = 572, 44.9%), followed by residents of the Southern governorate (n = 275, 21.6%). Patients aged 12-18 months (n = 192, 15.0%) represented the largest group in the study sample, followed by those aged 6-12 months (n = 162, 12.7%). Regarding the vaccination status of the enrolled patients, 187 (14.6%) were under the age of vaccination (less than 1 year), 233 patients received one dose of MCV, 713 (55.7%) patients received two doses, and 147 (11.5%) did not have evidence of vaccination. Of the total sample, 1044 (81.6%) had IgG-positive results (Table 1). Among these positive cases, 46 (4.4%) were classified as weakly positive.

**Table 1:** Measles immunoglobulin G results of the 1280 patients enrolled in the study.

Characteristics		Total patients (%)^a^	Immunoglobulin G–positive (%)^b^	95% Confidence interval	*P*-value^c^
Overall		1280 (100%)	1044 (81.6%)	(79.3-83.7%)	-
Sex	Female	556 (43.4%)	456 (82.0%)	(78.5-85.1%)	0.7
	Male	724 (56.6%)	588 (81.2%)	(78.1-84.0%)	
Nationality	Bahraini	1020 (79.7%)	843 (82.6%)	(80.2-84.9%)	0.047
	Non-Bahraini	260 (20.3%)	201 (77.3%)	(71.6-82.2%)	
Governorate	Capital	228 (17.8%)	188 (82.5%)	(76.8-87.0%)	0.7
	Muharraq	200 (15.6%)	167 (83.5%)	(77.5-88.2%)	
	Northern	572 (44.7%)	460 (80.4%)	(76.9-83.5%)	
	Southern	275 (21.5%)	227 (82.5%)	(77.4-86.7%)	
	Unknown	5 (0.4%)	2		
Age group	<6 months	19 (1.5%)	2 (10.5%)	(1.8-34.5%)	<0.001
	6 to <12 months	162 (12.7%)	8 (4.9%)	(2.3-9.8%)	
	12 to <18 months	192 (15.0%)	145 (75.5%)	(68.7-81.3%)	
	18 to <24 months	78 (6.1%)	73 (93.6%)	(85.0-97.6%)	
	2 to <3 years	125 (9.8%)	124 (99.2%)	(95.0-100.0%)	
	3 to <4 years	103 (8%)	102 (99.0%)	(93.9-99.9%)	
	4 to <5 years	137 (10.7%)	136 (99.3%)	(95.4-100.0%)	
	5 to <6 years	127 (9.9%)	126 (99.2%)	(95.0-100.0%)	
	6 to <7 years	90 (7%)	90 (100.0%)	(94.9-100.0%)	
	7 to <8 years	71 (5.5%)	69 (97.2%)	(89.3-99.5%)	
	8 to <9 years	46 (3.6%)	46 (100.0%)	(90.4-100.0%)	
	9 to <10 years	40 (3.1%)	37 (92.5%)	(78.5-98.0%)	
	10 to <15 years	40 (3.1%)	38 (95.0%)	(81.8-99.1%)	
	≥15 years	50 (3.9%)	48 (96.0%)	(85.1-99.3%)	
Measles, mumps, and rubella vaccination status	One dose	233 (18.2%)	202 (86.7%)	(81.5-90.6%)	<0.001
	Two doses	713 (55.7%)	710 (99.6%)	(98.7-99.9%)	
	Underage^d^	187 (14.6%)	11 (5.9%)	(3.1-10.6%)	
	Unvaccinated	147 (11.5%)	121 (82.3%)	(75.0-87.9%)	

a Column percentages.

b Row percentages.

c *P*-values calculated using the chi squared test, except for age group were the Fisher’s exact test was used.

d Underage was defined as patients below the age of 12 months.

### Measles IgG seroprevalence

The overall seroprevalence of measles IgG was 81.6% (95% CI: 79.3-83.7%). There was no difference in the seroprevalence between males (82.0%; 95% CI: 78.5-85.1%) and females (81.2%; 95% CI: 78.1-84.0%). Regarding the patient’s nationality, there was some evidence that the seropositivity rate differed between Bahraini and non-Bahraini populations, where 82.6% (95% CI: 80.2-84.9%) of the sampled Bahraini patients were seropositive compared with 77.3% (95% CI: 71.6-82.2%) among non-Bahrainis (*P* = 0.047). There was no evidence that the seropositivity rate differed by the residential governorate of the patient (*P* = 0.7) (Table 1).

**Figure 2:**
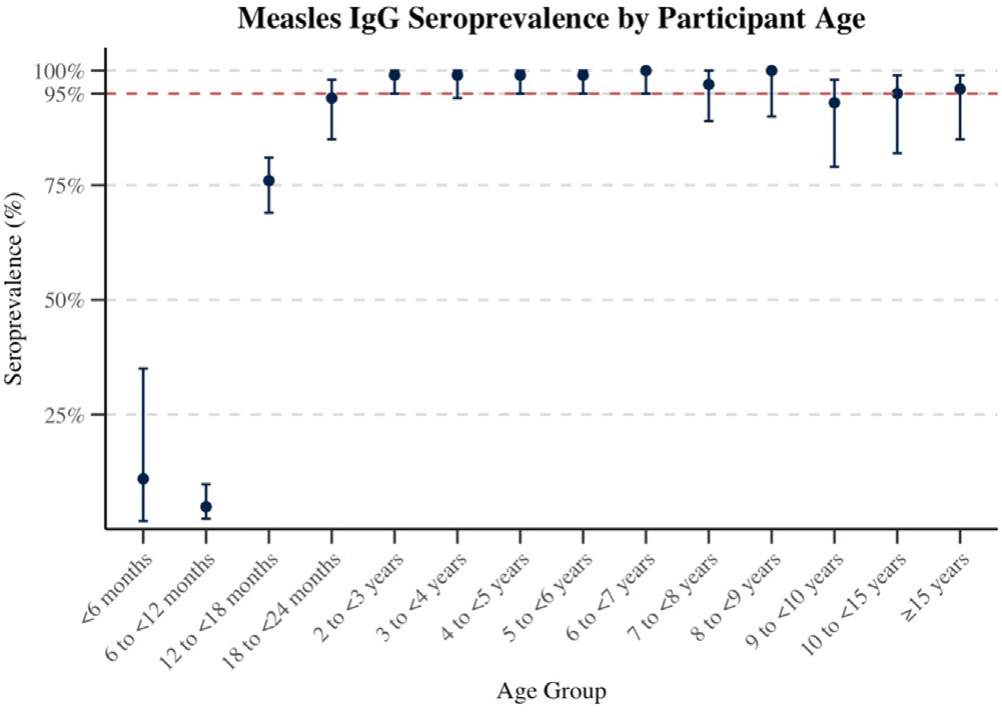
Measles IgG seroprevalence by participant age. Age-specific measles IgG seroprevalence among study participants (N = 1280) in Bahrain. Data points represent seroprevalence estimates with 95% confidence intervals for each age group. The dashed horizontal line indicates the 95% herd immunity threshold. The x-axis represents the age groups, whereas the y-axis indicates the IgG seroprevalence. Ig, immunoglobulin.

There was strong evidence of differences in seroprevalence by age (*P* <0.001). The seroprevalence rate among patients younger than 6 months was 10.5% (95% CI: 1.8-34.5%), dropping to 4.9% (95% CI: 2.3-9.8%) among those aged 6 to less than 12 months. The proportion increased markedly to 75.5% (95% CI: 68.7-81.3%) among patients aged 12 to less than 18 months and further rose to 93.6% (95% CI: 85.0-97.6%) among those aged 18 to less than 24 months. Seroprevalence remained consistently above 95% in older age groups, except for those aged 9 to less than 10 years (92.5%; 95% CI: 78.5-98.0%) (Table 1) (Figure 2).

Regarding vaccination status, individuals below the eligible age for vaccination had a seroprevalence of 5.9% (95% CI: 3.1-10.6%). Those who received one dose of MCV had a seroprevalence of 86.7% (95% CI: 81.5-90.6%), which increased to 99.6% (95% CI: 98.7-99.9%) among recipients of two doses. Patients without documented vaccination had a seroprevalence of 82.3% (95% CI: 75.0-87.9%), with strong evidence that seroprevalence differed by vaccination status (*P* <0.001).

### Age-adjusted measles IgG seroprevalence

**Figure 3:**
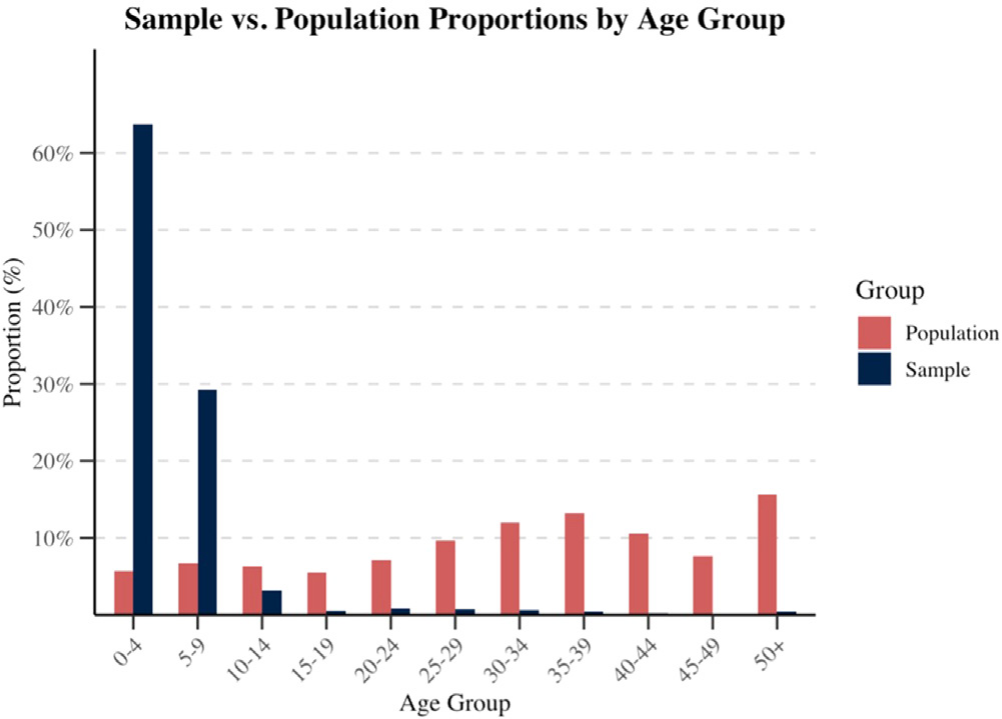
Study sample vs Bahrain population age structure. Comparison of age group distributions between the study sample and Bahrain’s 2023 population age structure.

To adjust for the over sampling of younger age groups and the under sampling of older age groups relative to Bahrain’s age structure, post-stratification weighting was applied (Figure 3). After adjusting for the age structure, the estimated seroprevalence of measles IgG was 94.3%, with a 95% CI ranging from 85.7-97.9%.

## Discussion

In our sample, the overall seroprevalence of measles IgG was 81.6% (95% CI: 79.3-83.7%). After adjusting for the age structure of Bahrain’s population, the estimated seroprevalence was estimated to be 94.3% (95% CI: 85.7-97.9%).

There was no evidence of a difference in seroprevalence when stratified by sex or governorate of residence. However, there was some evidence of a difference by nationality, with higher seroprevalence observed among Bahraini patients. There was strong evidence of a difference that seroprevalence differed by patients age group. The seroprevalence was lowest among infants aged under 6 months (10.5%) and among those aged 6-12 months (4.9%). Regarding vaccination, there was strong evidence of an association between vaccination status and measles IgG seroprevalence, with the highest seroprevalence observed among those who had received two doses of MCV.

This study had several strengths. Relying on the established national surveillance system enabled precise estimation of measles IgG seroprevalence, particularly, among younger patients. The use of surveillance data also ensured the inclusion of all patients who accessed primary and secondary care services in Bahrain across all four governorates, enhancing geographic representativeness. In addition, relying on routinely collected data minimized the risk of recall bias and reduced the extent of missing data. Also, it limited self-selection bias because all eligible notified patients were included in this analysis.

However, our study had also several limitations. The sample was derived from the national fever and rash surveillance system, which, although practical, and suitable for rapid estimation of measles IgG seroprevalence, introduced inherent sources of selection bias. First, younger age groups (<10 years) were over-represented compared with older individuals (>10 years), making the sample unrepresentative of Bahrain’s population age structure. Although post-stratification weighting was applied to correct for this imbalance, the estimates for older age groups remained less precise due to small sample sizes. Thus, the external validity of our findings should be verified in future, more comprehensive serosurveys. Second, the surveillance system may have disproportionately captured vaccinated individuals because some develop fever and rash post-vaccination, increasing their likelihood of being notified [25], which could lead to an overestimation of measles IgG seroprevalence. Third, restricting our sample to patients notified through the fever and rash surveillance system may have led to an overestimation of seroprevalence. These individuals may differ from the general population in their health-seeking behaviors, health awareness, and adherence to vaccination programs, which could overestimate the seroprevalence. Fourth, physicians’ decisions to notify suspected cases may have been influenced by patients’ previous vaccination status [19]. This may have introduced additional selection bias, potentially resulting in either over-representation or under-representation of vaccinated individuals, depending on the timing of vaccination.

Moreover, the assay used to detect measles IgG had a sensitivity of 96.6%, which may have led to the misclassification of some seropositive patients as seronegative, thus underestimating the seroprevalence and providing a less precise estimate [26]. Further, vaccination status was determined from electronic health records, which may be incomplete, particularly, for older patients. This could potentially overestimate seroprevalence among individuals with undocumented vaccination histories. Lastly, a set of requested tests were not performed, possibly due to patients not meeting the surveillance case definition or logistical constraints, such as reagent limitations. These reasons are unlikely to be related to patients’ underlying measles IgG status. Another set of tests were not performed due to insufficient blood volume, which may be associated with younger age; however, our primary estimate was adjusted for age. Overall, although this missingness reduced the precision of our estimates, it is less likely to affect the validity of our findings.

Comparing our findings with serosurvey data from other countries highlights regional variations in measles immunity. Our observed age-adjusted seropositivity proportion was slightly higher than the estimated seroprevalence reported in Saudi Arabia, where a serosurvey conducted in Madinah among vaccinated and previously infected individuals found a seroprevalence of approximately 92% [27]. In Oman, a serosurvey using anonymized samples reported a lower estimate of 84% [28]. A study conducted in Madagascar, which similarly relied on patients notified through the fever and rash surveillance system, estimated an overall seroprevalence of 83.2%, which is lower than our estimated seroprevalence. This could be reflecting Madagascar’s lower vaccination coverage [19]. Similarly, a study in Hunan, China estimated seroprevalence at 80.9% [29]. In Mexico, a population-based serosurvey reported a substantially lower seroprevalence of 61.1%, as measured by enzyme-linked immunosorbent assay [30].

Examining age-specific seroprevalence, a study conducted in Iran reported IgG positivity of 38% in children aged under 6 months, 19% in those aged 6-12 months, and 60% in those aged 12-18 months [31]. This trend was similar to what we observed in our data, although we found the seroprevalence to be lower in those aged less than 12 months and higher in those aged 12-18 months.

The estimated age-adjusted seroprevalence of measles IgG was 94.3% (95% CI: 85.7-97.9%), which falls within the range required to achieve herd immunity and interrupt measles transmission [8]. Sustained efforts to maintain and strengthen vaccination coverage, as well as continued surveillance, are essential to maintain the proportion of susceptible individuals below the threshold.

In addition, non-Bahraini nationals demonstrated lower seroprevalence than Bahraini nationals. The lower seroprevalence observed among younger non-Bahrainis may be influenced by differences in vaccination history and health care system utilization. For adult non-nationals, who constitute approximately 87.5% of the non-Bahraini population, it could reflect lower vaccination coverage and immunogenicity of vaccines used in their countries of origin, as well as differences in national immunization policies [12,32]. For example, India only introduced a two-dose measles schedule in 2010 [33]. Although all school students are required to complete the recommended vaccine doses, the immunity gap observed among younger non-national children may indicate some challenges with health care system integration upon arrival [34]. These differences may also be due to varying health-seeking behaviors and vaccination uptake across non-national communities.

We also found that measles IgG seroprevalence was low among children aged 6-12 months, which is likely due to the wanning of maternal antibodies. Historically, maternal immunity was thought to protect infants for up to 12 months. However, studies in measles-eliminated settings show that maternal antibodies decline more rapidly, particularly, among vaccinated mothers compared with those naturally infected [31,35,36]. The reduced seroprevalence in infants aged 0-6 months likely reflects this early loss of passive immunity, especially in populations where maternal immunity is vaccine-induced [37]. The further decline among infants aged 6-12 months creates a window of susceptibility, increasing their risk of measles infection [37,38]. Thus, infants under 12 months may be particularly vulnerable during outbreaks [39]. To address this gap, the WHO recommends considering a supplementary vaccine dose for infants older than 6 months during outbreaks as part of an intensified outbreak control measure [7]. Therefore, a supplementary dose for infants aged 6-12 months could be considered during future outbreaks, although this must be balanced against logistical requirements and potential effects on the routine schedule. Among children aged 12-18 months, seroprevalence (75.5%) was lower than in older age groups, likely reflecting that children in this age group have received at most one dose, with the second dose administered at 18 months.

Future studies are required to explore health-seeking behaviors among the population to better understand potential factors contributing to vaccine hesitancy or non-adherence. Finally, validating the findings of this study using formal methods, such as community based serosurveys, is required.

## Conclusion

In conclusion, the estimated seroprevalence of measles IgG was 94.3% (95% CI: 85.7-97.9%) after adjusting for the country’s age structure. We found some evidence that seroprevalence differed by patients’ nationality and strong evidence that it differed by age group and vaccination status. In addition, individuals under 12 months had the lowest seroprevalence. To maintain immunity levels above the herd immunity threshold, sustained vaccination and surveillance efforts are necessary. Moreover, efforts should be made to maintain high vaccine coverage among non-Bahraini population through school based and supplementary immunization activities. Further research is required to validate these findings, with future studies also exploring health-seeking behaviors and beliefs to guide targeted health awareness and education efforts.

## Declaration of competing interest

The authors have no competing interests to declare.

## Appendix Supplementary materials

Image, application 1
