# Supplementary material for: Seroprevalence of measles immunoglobulin G antibodies in Bahrain: A cross-sectional study using fever and rash surveillance data (2021-2025)
Source: IJID Reg. 2026 Mar 7;19:100871. doi: 10.1016/j.ijregi.2026.100871 (PMC13054100; doi:10.1016/j.ijregi.2026.100871)
Supplement: Supplementary file 1 [file mmc1.pdf]

Ref. No. : AUPH-2025-H-000 81

Date : 22 July 2025

To : Dr. Adel Al Sayyad

Public Health Advisor

Ministry of Health

Co- investigators: Dr. Basma Al Safar, Dr. Afaf Mohamed, Dr. Ebrahim Matar, Dr Zahra Zabar, Wafa Fawzi and Mona Husain.

Subject: Letter of Conditional Approval for Research Proposal.

"Seroprevalence of Measles IgG Antibodies in Bahrain: A Cross-Sectional Study Using Fever and Rash Surveillance Data (2021–2025)"

Dear Dr. Adel,

Thank you for submitting the above research Protocol proposal, which was considered by members of the Health Research Committee in Ministry of health.

Please note that this approval is also subject to the following conditions:

1. We expect that the study will begin within 6 months of the date of this approval.
2. Approval from a Health research committee does not automatically imply that the researcher is granted access to data, medical records, or biological samples from MOH healthcare facilities. Researchers must seek permission and follow procedures as dictated by the concerned departments after presenting them with a valid MOH approval letter.
3. Any significant change, which occurs in connection with this study and / or which may alter its ethical consideration, must be reported immediately to the Health Research Committee.
4. This approval is valid for up to 1 year from the date of approval. If the study extends beyond this date, a progress report must be sent to the Health Research Committee to renew the approval.
5. The Health Research Committee must be informed when the research has been completed, and a copy of the final research report must be submitted to our records.

Yours sincerely,

Dr. Samya Ali Bahram

Assistant Undersecretary for Public Health

Chief of Research Committee at Ministry of Health
